# Your face looks the same as before, only prettier: The facial skin homogeneity effects on face change detection and facial attractiveness perception

**DOI:** 10.3389/fpsyg.2022.935347

**Published:** 2022-11-02

**Authors:** Yu-Hao P. Sun, Xiaohui Zhang, Ningyan Lu, Jing Li, Zhe Wang

**Affiliations:** ^1^Department of Psychology, Zhejiang Sci-Tech University, Zhejiang, China; ^2^Institute of Dermatology, Chinese Academy of Medical Sciences and Peking Union Medical College, Beijing, China

**Keywords:** facial attractiveness, face change detection, facial skin homogeneity effect, face perception, psychophysical tasks

## Abstract

Previous studies suggested that (1) facial attractiveness perception can be increased with facial skin homogeneity improving; and (2) human’s facial change detection increases along with facial skin homogeneity increases. However, it’s unknown whether a face can be perceived prettier than it did before while still being considered as physically the same. It is possible that these two kinds of cognitive-aesthetic processing may have separate mathematical functions in psychophysical studies. In other words, human’s facial attractiveness differentiation may be more sensitive than facial change detection. In this current study, we explored the above questions. Using three types of psychophysical techniques to manipulate facial skin homogeneity, we measured how participants’ sensitivity to facial skin homogeneity and attractiveness change. Results showed a linear function curve for facial physical change detection and a logarithmic function curve was drawn in the forced-choice technique, which was the most sensitive one, indicating that participants can judge a face prettier than before without being aware of it has physically changed. Besides, two linear function curves were shown in the same/different technique and a rating technique. Taken together, this current study revealed that facial attractiveness can be enhanced and discriminated by improving facial skin homogeneity, without being realized by people with conscious awareness that the face has been changed.

## Introduction

Facial attractiveness has an important impact on various aspects of social life. By investigating the “What is beautiful is good” effect, researchers have shown that attractive individuals are perceived as having a spate of positive traits. In mock scenes, attractive candidates have a higher estimated probability of getting an interview (Shtudiner, 2020). And people are more tolerant to unethical behaviors from attractive individuals (Shtudiner and Klein, 2020). In real life, individuals’ beauty benefits them in marriage, education, labor market and increases their happiness (Hamermesh and Abrevaya, 2013). Attractiveness has significant positive impacts on a professor’s promotion and career success (Liu Y. et al., 2022). Attractive individuals may be favored in the college admissions process (Ong, 2022) and get more fringe benefits at work (Dilmaghani, 2020). As stated by Langlois et al. (2000): “beauty is more than just skin-deep” (p.404).

Perfect facial skin is one of the most desirable traits for the attractive face (Fink et al., 2001). Skin homogeneity has been proved a key property to facial attractiveness perception. Fink et al. (2001) extracted the texture homogeneity, contrast features and color parameters of 18–25-year-old female faces through computer technology and found that attractiveness rating score of faces made by male participants related positively to parameters of skin texture and color homogeneity. After that, many studies replicated that finding. They found that facial skin texture and color homogeneity were both positively associated with facial attractiveness rating in different ages for the female (Color: Fink et al., 2006; Fink et al., 2008; Texture and Color: Fink and Matts, 2008; Samson et al., 2010b,^2011^).

Facial homogeneity is typically described as facial skin’s smoothness in texture or facial skin color distribution (Samson et al., 2010b,^2011^). For example, Little et al. (2011) argued that people can feel attractiveness and healthy status from one’s facial skin. Even the forehead with a minor skin region provided useful information for the attractiveness of the whole face (Liu C. H. et al., 2022). Positive correlations between facial skin health rating and facial attractiveness rating have been found by many researchers (e.g., Jones et al., 2004; Whitehead et al., 2012; Lu et al., 2021). The faces with radiant and smooth skin can enhance perceptions of health and attractiveness and convey various positive impressions to the observers, while the faces with skin blemishes convey the impression of unhealthy and incompetent (Jaeger et al., 2018; Ikeda et al., 2021; Sakano et al., 2021).

Male faces perception showed similar effects. For example, Fink et al. (2012) used an image segmentation algorithm to objectively analyze the homogeneity of skin color on male faces. They found that male faces with homogenous skin color (with even melanin and hemoglobin distribution) were perceived as healthier and more attractive. After that, Fink et al. (2018) manipulated facial texture by removing face wrinkles or skin color features (e.g., dark spots, hyperpigmentation, telangiectasias) from the faces of men aged 40–70. They found that faces with the evener texture were perceived as younger, healthier, and more attractive than their original versions.

Studies on Asian faces showed similar results to studies on Caucasian faces. Tan et al. (2018) found that homogenous skin texture and skin color distribution could predict the health grade of Malaysian Chinese faces through wavelet analysis measurements. A study on Chinese faces also found that the faces after removing dark spots, wrinkles, and dark circles were more attractive than their original versions (Porcheron et al., 2014). In conclusion, the homogenous facial skin positively affects facial attractiveness, which is consistent across age, gender, and ethnicity.

Are individuals able to find a face that is more attractive without noticing it has physically changed from the last look? This is an intriguing question. If yes, it means that the human perceptual system can assess facial attractiveness when the facial skin homogeneity is altered but the face physical change detection is not involved in such cognitive-aesthetic processing. It sounds like a paradox, but some studies showed it is possible to see a dissociation between these two kinds of psychological judgments. For example, using psychophysical methods, Re et al. (2011) detected participants’ thresholds to face skin redness sensation change and facial attractiveness change. Their findings suggested that participants were more sensitive to changes in facial skin than to changes in facial attractiveness, since the threshold to redness feeling was lower than that to face attractiveness.

In this current study, we aimed to explore participants’ difference detection sensitivity and their attractiveness change perception to faces which were manipulated in their skin homogeneity. We conducted four experiments. In Experiment 1, participants were asked to make the same/different judgment to two face photographs from the same person in each trial. These two face photographs were an original version face (0% beautification) as a standard stimulus and one version which was selected randomly from its 4 beautification level versions (0, 30, 45, and 100%) by a facial skin beautification algorithm. In this way, participants’ detection to facial physical changes can be measured. In Experiments 2A, 2B, and 2C, we used psychophysical techniques on three levels of sensitivity to measure participants’ facial attractiveness perception. In Experiment 2A, we used a forced-choice technique. It is the most sensitive one. Participants were asked to make a forced choice (“Which face is prettier?”) between an original version and a skin-beautified version of a face. In Experiment 2B, we also used the same/different judgment for the two faces which was similar to Experiment 1. In Experiment 2C, we used a rating technique on psychometric level. Participants were asked to rate the facial attractiveness degree for each face on a six-point rating scale, which was presumed as the most insensitive in all these three techniques.

Although attractiveness is affected by more objective characteristics, such as facial symmetry (Grammer and Thornhill, 1994), the subjective attractiveness rating is widely used in economics, marketing, and psychology studies (Wilson and Eckel, 2006; Andreoni and Petrie, 2008; Deryugina and Shurchkov, 2015; Shapir and Shtudiner, 2021; Ruffle et al., 2022). Evidence shows that there is substantial agreement between the subjective attractiveness rating and incentivized coordination game attractiveness measure (a more objective evaluator-derived measure, see, Babin et al., 2019). It is also highly correlated with the most objective computer-based assessment (Zhao et al., 2020). The attractiveness rating seems to be a reliable indicator of facial attractiveness. We used this technique together with the same/different detection and force-choice task on attractiveness measurement in Experiment 2.

The basic hypothesis we would test in this study is that facial attractiveness perception could be more sensitive than face physical change detection. The rationale for the two experiments is as followed: Since the two tasks (i.e., physical same/different detection vs. attractiveness perception, respectively) in Experiments 1 and 2 asked participants to make judgments along two different psychological dimensions (i.e., physical vs. pretty), they should be involved in separate psychological mechanisms. It is reasonable to test and measure how much participants’ sensitivity in physical change detection is different from the sensitivity to facial attractiveness change. We expected to observe two or three dissociated mathematical function curves in Experiments 1, 2A, and 2B. Besides, we also explored the capacity of a typical psychometric rating scale technique in Experiment 2C by comparing this technique’s sensitivity to facial attractiveness in Experiment 2C with the two regular psychophysical techniques in Experiments 2A and 2B (The reason why we chose that two techniques was partly because that two techniques have shown good sensitivity in prior studies and our pilot test).

Specifically, we had three predictions. (1) According to the evidence from previous studies, the facial skin homogeneity effects on facial attractiveness should increase and, slower and slower, reach a certain level, gradually. (2) If participants are more sensitive to facial attractiveness than physical change, they should perceive a beautified face as more attractive without detecting that this face is different from its original version. (3) On the contrary, if participants are equally or less sensitive to facial attractiveness change than to facial physical change in facial skin homogeneity, they should easily judge the beautified faces as physically different from their original versions, with or without perceiving it as more attractive.

## Experiment 1: Detecting facial physical change

We examined participants’ detection sensitivity to face physical change by manipulating faces’ skin homogeneity. Participants were asked to make same/different judgments between a skin-beautified face and its original version in each trial.

### Participants

Thirty-six undergraduates (mean age = 20.7 years, SD = 2.55, age range: 18–27 years; 18 males, 18 females) participated in Experiment 1. The required sample size calculated by G-Power (v3.1) is 9, for a one-factor four-level within-subject design experiment detecting this effect size at the 0.05 alpha level with 95% power.

All participants had normal or corrected-to-normal vision. They were unfamiliar with the face used in the experiment. They participated voluntarily, signed the informed consent before the experiment began, and got paid after they completed it.

### Materials

Fourteen Chinese face photographs (aged 17–18, seven women and seven men) were selected from our photograph database. The models kept neutral expressions without jewelry, glasses, or makeup on their faces during image acquisition. To reduce the biases caused by distinctive facial skin, we selected the 14 images based on the following criteria: No overly pale or dark skin color and no severe skin blemishes or pimples on the face. We equilibrated the skin condition of male and female faces concurrently. Male and female faces had a similar degree of skin color, radiance, and blemishes. We unified all images to be 390 pixels in width using Adobe Photoshop. The height of the images had different pixels because of the different face shapes.

**FIGURE 1:**
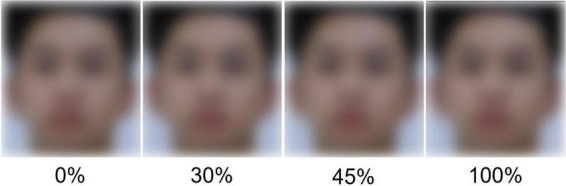
Sample images of the faces on different beautification levels. Facial skin homogeneity increases sequentially from left to right. The leftmost image is the original face with 0% beautification, and the remaining three images are its variant versions with 30, 45, and 100% level beautification.

Using a facial skin beautification algorithm (BeautyCam, v.10.0.70, 2021, Meitu Inc., Xiamen, China), we homogenize the skin texture and color of the original face images. With this manipulation, we made a set of 56 faces with four levels of skin beautification (0, 30, 45, and 100%). The 0% beautification level was the baseline at which the beautified face was the same as the original face. We did a few pilots. We compared the original face and beautified images on several levels. We selected the 30% beautification level as the first level higher than 0% because: (1) in the same/different technique (e.g., in Experiment 1), the faces beautified lower than 30% level looked to us very similar to their original versions (that was also the reason why we did consider but ignored inserting a 15% level); (2) in the forced-choice technique (e.g., in Experiment 2B), 30% level beautification has a response rate higher than chance level and we roughly predicted that data on 30% level might be approximately near the flex point of the logarithmic curve (yes we roughly predicted the curve might be fitted well to a logarithmic function). Then, we selected the 45% level because of two similar reasons: (1) in the same/different technique, 15% (i.e., from 30 to 45%) is a pace large enough to see a significant difference; and (2) in the forced-choice technique, 45% and 30% levels together better than a single 30%-level data point in the curve. Finally, we selected the 100% level beautification, the upper limit of the operation in the software, as the highest manipulation. Taken together, by a pilot study and visual inspection, we decided to set the 30% beautification as the low-level, the 45% as the medium-level, and the 100% as high-level beautification. Figure 1 shows an example of the faces on different beautification levels.

### Task and procedure

The participants performed the experiment in a quiet environment with sufficient light. The experiments were carried out by a computer with a screen resolution of 1024 × 768. The experimental program was written with E-prime 2.0.

Participants were asked to make the same/different judgment to two faces, physically, in each trial. In instructions, we informed participants the two faces belonged to the same person. We did not emphasize the changes of skin. Because we expected that they would view the whole face rather than just the skin in the task, as they would do in everyday life. We also set practice trials to check participants to make their judgment based on face physical changes. The “same” response (pressing the number key “1”) meant that the two faces were judged to be the same, while the “different” response (pressing the number key “2”) meant that the two faces were considered different. These two faces were randomly ordered. One was the original (0% beautification) and the other was randomly selected from the four versions (the original and that on the three beautification levels: 30, 45, and 100%). To ensure that the participants accurately understand the experimental requirements, practical trials were arranged before the formal experiment trials. The face images that appeared in the practice phase did not appear in the formal experimental phase.

**FIGURE 2:**
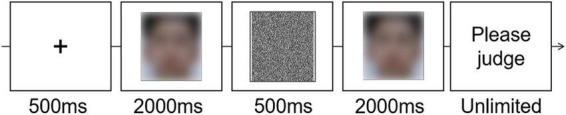
The trial flow of Experiment 1.

The flow of a trial is shown in Figure 2. Each trial started with a fixation cross (500 ms) on the screen center. Then, a face was displayed (2,000 ms), followed by a visual mask consisting of black dots covering the whole face image (500 ms). After that, the second face was displayed (2,000 ms). At the same time, participants were asked to make a “same” or “different” judgment by pressing one key. If the response had not been recorded when the second face disappeared, the words “Please judge” would be displayed on the screen until participants pressed a key within the two.

All participants completed two blocks of male and female faces in a random order. The two faces within a face pair trial were presented in a balanced order. In total, each face pair was presented twice, making a total of 112 trials.

### Results and discussion

**FIGURE 3:**
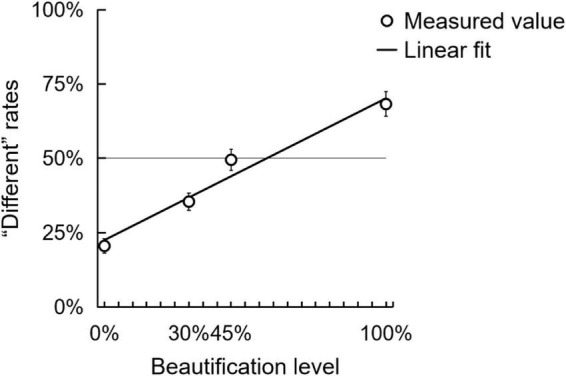
Detecting facial physical change (Experiment 1). The chart shows the “different” rates and standard errors for each beautification level. The oblique line represents the fitted linear function.

We recorded the rates of “different” judgments in total. The average “different” rate for each beautification level is shown in Figure 3.

First, a repeated-measures ANOVA was conducted on “different” rates with the facial skin beautification level (0, 30, 45, and 100%) as a within-subjects factor. The main effect of beautification level was significant, [*F*(3,33) = 52.640, *p* < 0.001, η*_*p*_*^2^ = 0.827], indicating that the more homogenous the beautified skin, the better participants differentiated the original version and the beautified version of a face.

Secondly, on the zero beautification (i.e., 0%) level, the “different” rate was 20.54% (±14.1%), not 0%. This indicated participants’ response on the objective baseline, which was in subconsciousness. Also, because the chance level (50%) is the test value indicating a subjective threshold in conscious awareness, the “different” rates on 30 and 45% beautification levels were significantly higher than 20.54% baseline but not significantly higher that chance level (50%), showing that participants still not realize these two versions of faces different from the original one with conscious awareness.

To quantitatively describe how the participants perceived the beautified vs. original version not as the same, we performed one-sample *t*-tests comparing the “different” rate on each positive beautification level to the chance level (50%). Results showed that the rate (20.54 ± 14.1%) on 0% beautification level and the rate (35.42 ± 16.9%) on 30% beautification level were significantly lower than chance level (50%) [0%: *t*(35) = −12.547, *p* < 0.001, *d* = 2.091; 30%: *t*(35) = −5.187, *p* < 0.001, *d* = 0.864]. It indicated that participants refused to regard these two versions of faces as different with a conscious awareness. The “different” rate (49.50 ± 21.4%) on 45% beautification was not significantly different from 50%, [*t*(35) = −0.139, *p* = 0.890, *d* = 0.023], indicating that participants cannot tell them apart, yet. The “different” rate (68.25 ± 24.4%) on 100% beautification level was significantly higher than the chance level, [*t*(35) = 4.475, *p* < 0.001, *d* = 0.746], indicating participants can find them as two.

Finally, a linear fit was performed. Figure 3 shows that a linear function fits the points well, [*y* = 0.477×*x* + 0.225, *F*(1,2) = 59.686, *p* = 0.016, *R*^2^ = 0.968].

Taken together, these results showed that participants were unable to distinguish between the original and beautified versions of a face unless the facial skin became strongly heterogeneous (i.e., it must be beautified significantly higher than 45% level, quantitatively).

## Experiment 2: Perceiving facial attractiveness change

### Participants

We recruited three groups of participants for three sub-experiments: 2A, 2B, and 2C. Twenty-one undergraduates (mean age = 19.14 years, SD = 1.39, age range: 18–22 years; nine males, 12 females) were in Experiment 2A. Eighteen undergraduates (mean age = 18.33 years, SD = 1.24, age range: 17–22 years; nine males, nine females) were in Experiment 2B. Seventeen undergraduates (mean age = 21.82 years, SD = 2.04, age range: 19–25 years; eight males, nine females) were in Experiment 2C.

All participants had normal or corrected-to-normal vision. They were unfamiliar with the face images used in the experiments by their self-reports. They participated voluntarily, signed the informed consent at the beginning of the experiment, and got paid after they had completed it.

### Materials

The stimuli in Experiment 2 were the same as in Experiment 1.

### Procedure

Participants’ facial attractiveness perception was measured in three types of psychophysical techniques.

**FIGURE 4:**
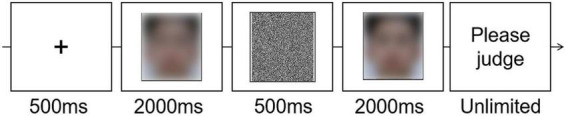
The trial flow of Experiments 2A and 2B.

In Experiment 2A, we used a forced-choice technique (tech A) for participants group A. Participants were asked to make a forced choice on the attractiveness degree between a skin-beautified face (randomly selected from 0, 30, 45, and 100% ones) and its original version (0% beautification). The rest of the process was similar to that of Experiment 1, including that the two faces were presented one by one, with a random sequence. The flow of a trial is shown in Figure 4.

In Experiment 2B, we used the same/different technique (tech B) for participants in group B. They were asked to make the same/different judgment on the attractiveness degree between a skin-beautified face (0, 30, 45, and 100%) and its original version (0% beautification). The “same” response indicated the two faces were judged as equally attractive. The rest of the process was similar to that of Experiment 2A. The flow of a trial is shown in Figure 4.

In Experiment 2C, we used a rating scale technique (tech C) for participants in group C. Participants were asked to rate the facial attractiveness degree for each face on a six-point rating scale (ranging from 1 to 6, representing from extremely low attractiveness to extremely high attractiveness). Each face was presented for unlimited time until participants pressed a number key.

Each group of participants completed two blocks of male and female faces in a random order. Each version of the beautified face was presented twice, in a random order. In total, 112 trials were presented during Experiments 2A, 2B, or 2C.

### Results and discussion

We organized Experiments 2A, 2B, and 2C’s data in one chart and performed statistics in three ways. For Experiment 2A, we recorded the rates at which beautified faces were considered more attractive, denoted as the “more attractive” rates. At the 0% beautification level, the two faces compared were physically the same. Thus, we set the choosing rate as 50%.

A repeated-measures ANOVA was conducted on “more attractive” rates with the facial skin beautification level (0, 30, 45, and 100%) as within-subjects factor. The main effect of beautification level was significant, [*F*(3,18) = 91.052, *p* < 0.001, η*_*p*_*^2^ = 0.938], indicating that the more homogenous the facial skin, the more attractive the face was perceived. We set the base rate as the chance level (50%). It is the probability that participants chose either of the two original faces in the baseline condition (0% beautification). The ratio on three levels (30, 45, and 100%) was significantly higher than the chance level (50%).

**FIGURE 5:**
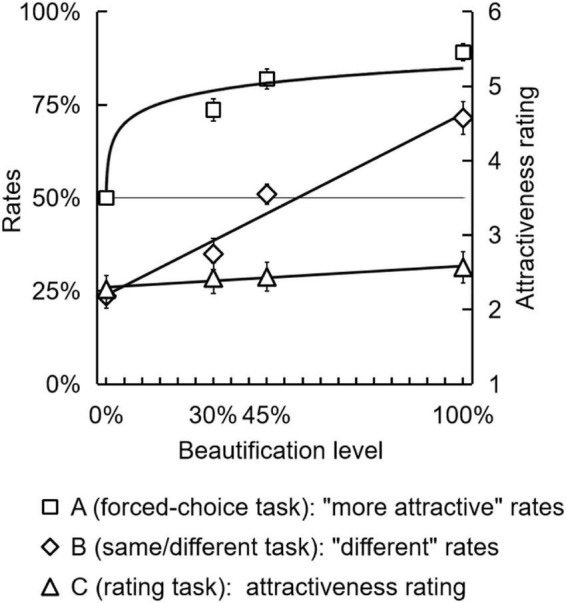
Perceiving facial attractiveness change (Experiment 2). The “more attractive” rates in Experiment 2A, the “different” rates in Experiment 2B, and the attractiveness rating in Experiment 2C were shown. The black lines represented the fitted functions.

We performed a logarithmic fit and a linear fit to the average “more attractive” rates for each beautification level in order to investigate the tendency of the “more attractive” rate to increase with skin homogeneity (We replaced the beautification 0% by 0.1% in the *x*-axis because the *x*-value of a logarithmic function could not be 0). Results revealed a logarithmic function (see Figure 5) [*y* = 0.052 × ln(*x*) + 0.854, *F*(1,2) = 36.803, *p* = 0.026, *R*^2^ = 0.948], that was consistent with Fechner’s law (Fechner, 1860) in sensation. Additionally, the outcome of the linear fit was not significant [*F*(1,2) = 8.080, *p* = 0.105]. We calculated the Akaike information criterion (AIC) of the two fitted functions to determine which function matched the data more closely (Akaike, 1974). The logarithmic function has a better fit since its AIC value (−10.000) is lower than the linear function’s (−7.486).

For Experiment 2B, we recorded the rates of participants making “different” judgments in the total judgments, denoted as the “different” rates in Figure 5. A repeated-measures ANOVA was conducted on “different” rates with the facial skin beautification level (0, 30, 45, and 100%) as the within-subjects factor. The main effect of beautification level was significant [*F*(3,15) = 23.956, *p* < 0.001, η*_*p*_*^2^ = 0.827], indicating that the more homogenous skin a face had, it would be judged as the higher “different” rate (0%: 23.65 ± 13.3%; 30%: 34.92 ± 17.3%; 45%: 50.99 ± 11.0%; 100%: 71.43 ± 18.1%). To explore the tendency of the “different” rates changing with the increase of the skin homogeneity, we performed a linear fit to the average “different” rates. The result showed almost an entirely linear function [*y* = 0.487 × *x* + 0.239, *F*(1, 2) = 60.862, *p* = 0.016, *R*^2^ = 0.968], showing that the stronger beautification, the higher “different” rate was there by the participants.

For Experiment 2C, the average attractiveness rating score for each beautification level were calculated and presented in Figure 5. A repeated-measures ANOVA was conducted on attractiveness rating scores with the facial skin beautification level as the within-subjects factor. The main effect of beautification was significant, [*F*(3, 14) = 8.114, *p* = 0.002, η*_*p*_*^2^ = 0.635]. To explore the tendency of the attractiveness rating changing with increasing skin homogeneity, we performed a linear fit to the attractiveness rating. The result showed a linear function increased very weakly (almost parallel to the *x*-axis), [*y* = 0.285 × *x* + 2.300, *F*(1, 2) = 34.936, *p* = 0.027, *R*^2^ = 0.946], suggesting the rating scale technique is not sensitive enough for measuring subtle facial attractiveness change in facial aesthetic perception studies.

### Detecting facial physical change vs. perceiving facial attractiveness change

**FIGURE 6:**
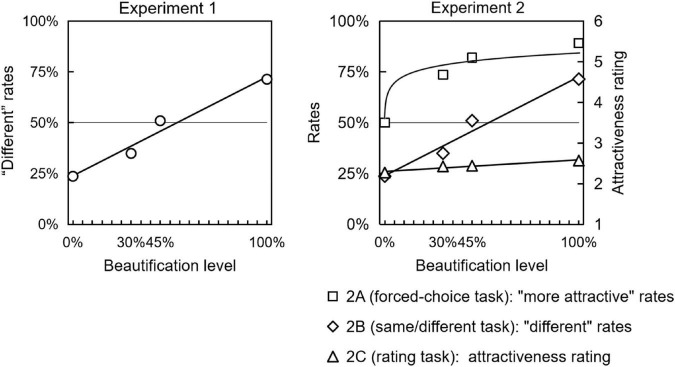
Detecting facial physical change in Experiment 1 and perceiving facial attractiveness change in Experiment 2.

The results of Experiment 1 (detecting facial physical change) and Experiment 2 (perceiving facial attractiveness change) are shown in Figure 6. In Experiment 1, results showed that the “different” judgment rate fitted a linear function. Only when facial skin homogeneity beautification on 100% level, the rate was significantly higher than the chance level. On this level, participants made judgments with a conscious awareness. However, In Experiment 2A, on all the 30, 45, and 100% beautifications, the ratio of “more beautiful” judgments was significantly higher than the chance level. The logarithmic curve fitted the data probed by the face attractiveness forced-choice technique.

Participants’ correct response to the prettier face in each trial increased so rapidly from 0 to 30% beautification. We can see the rate at the 30% level in Experiment 2A was higher than the rate at the 100% level in Experiment 1. These results strongly indicated that people perceived that the face with more homogeneous skin was more attractive.

We should note that only four levels of facial skin homogeneity in this study were manipulated. It is a little bit rough for curve-fitting algorithms. We agree that 0, 30, 45, and 100% were not as typical as 0, 25, 50, and 100% setting which we can see in many literatures. We also regarded 0, 15, 30, 45, and 100% better than our current version. A more exact curve may be fitted in a study with more, fine-grained quantitative data points. However, consider the pace setting in this current study make our hypotheses test went well, and the response rates on the 45%-level in the same/different technique were so close to 50%, we agree to regard the 45% level as a qualified medium-level manipulation in this current study.

We also noticed that, in Experiment 2B, the fitting function showed a similar linear pattern to that in Experiment 1. The data and indication from the comparison of “logarithm function vs. linear function” above were solid, although it was possible that similar same/different techniques evoked such two lines much close.

Taken together, these results suggested that participants’ awareness of facial attractiveness can be more sensitive than that to whole-face physical changes.

To explore gender differences, we performed a repeated-measures ANOVA for each experiment with the beautification level and face gender as within-subjects factors and the participant gender as a between-subjects factor. The main effect of beautification level was significant in four experiments. In addition, the following effects are significant.

In Experiment 1, the main effect of face gender was significant [*F*(1,34) = 8.620, *p* = 0.006, η*_*p*_*^2^ = 0.202], showing that the “different” rate of female faces (45.93%) was higher than that of male faces (40.92%). In Experiment 2A, the interaction effect of beautification level and participant gender is significant [*F*(2,18) = 6.837, *p* = 0.006, η*_*p*_*^2^ = 0.432]. The “more attractive” rate of women was significantly higher than that of men at the 45% beautification level (*p* = 0.012), and there was no significant difference between the two at other beautification levels. In Experiment 2B, the main effect of face gender was significant [*F*(1,16) = 12.090, *p* = 0.003, η*_*p*_*^2^ = 0.430], showing that the “different” rate of female faces (50.04%) was higher than that of male faces (39.75%). No other main effects or interactions were significant.

In Experiments 1 and 2B, participants were better at distinguishing the beautified versions from the original versions of the female faces than the male faces, indicating that facial skin beautification was more effective for women. It is also suggested that the effect of visible facial skin condition on attractiveness is greater in female skin than in male skin (Samson et al., 2010a). Only female faces, no male ones, were found to benefit from skin smoothing in terms of attractiveness (Jaeger et al., 2018). Homogeneity in facial skin is the key factor for the youthful and healthy skin appearance of women (Fink et al., 2017; Lee et al., 2019), which is positively correlated with attractiveness (Otaka et al., 2019).

Participant gender differences were observed only at the 45% level in Experiment 2A. There was evidence that women performed better in the recognition of minor modifications in facial skin than men (Samson et al., 2010b). This difference may be explained by the fact that the main consumers of cosmetic and facial beauty treatments are women (Jin et al., 2022; Mohammed et al., 2022), making them to be more sensitive to facial skin conditions. Male and female judgments of male and female faces were mostly consistent in this study. Although there is evidence that men and women have different preferences for same-sex and opposite-sex faces (Little et al., 2008), the perceptions of attractiveness are generally suggested to be consistent across genders (Rhodes, 2006).

To test whether the results were reliable when the number of participants reaches 9, we divided the participants into two groups. Group 1 contained nine participants and Group 2 contained the rest of them. A repeated-measures ANOVA was performed with the Group as the between-subject factor and the beautification level as the within-subject factor. The main effects of Group were not significant, [Experiment 1: *F*(1,34) = 0.141, *p* = 0.710; Experiment 2A: *F*(1,19) = 0.046, Experiment 2B: *p* = 0.833; *F*(1,16) = 0.255, *p* = 0.621; Experiment 2C: *F*(1,15) = 0.006, *p* = 0.939], respectively, showing that with participants fewer than 20, or even 9, the results could be robust enough.

Finally, since the faces were repeated presented in every experiment in this current study, we divided the total trials in each experiment (i.e., Experiments 1, 2A, 2B, and 2C) into two halves: the Early halves and the Later halves. We compared these pairs by performing a repeated-measures ANOVA with Early vs. Later and the four beautification levels as within-subject factors. Results showed that the “Early vs. Later” main effects were not significant for any of the four experiments [Experiment 1: *F*(1,35) = 0.210, *p* = 0.650; Experiment 2A: *F*(1,20) = 0.104, *p* = 0.750; Experiment 2B: *F*(1,17) = 0.063, *p* = 0.804; Experiment 2C: *F*(1,16) = 0.052, *p* = 0.822], respectively. No other significant difference was found for beautification or interactions analysis, either. We draw a conclusion that repetition did not affect facial attractiveness in this current study.

## General discussion

In the current study, we explored participants’ face physical changes detection and attractiveness perception and had three findings. First, we found participants’ detection to facial physical change in skin homogeneity followed a linear function. Secondly, we found that three psychophysical techniques were different in sensitivity on measuring participants’ facial attractiveness perception. Thirdly, by the most sensitive psychophysical technique, we found that participants’ facial attractiveness perception to facial skin homogeneity followed a logarithmic function. Convergently, we revealed that people may perceive a beautified face to be more attractive without detecting this face as physically different from its original version, in a short interval of facial skin homogeneity change range.

Our findings showed that people’s judgment of facial physical change and attractiveness change was different with the manipulation of facial skin homogeneity. For low-level beautification, participants tended to think that the original faces were the same as their beautified faces, but the beautified faces were more attractive. This suggests that a slight increase in skin homogeneity is not enough to make people feel that the face has changed physically, but they can find that the face is more attractive. Furthermore, as the homogeneity of the face skin increases, the rate at which the original face and the beautified face are considered to be different also increases linearly accordingly (Experiment 1). However, the objective change of facial skin homogeneity and people’s psychological perception of face attractiveness are not simple linear relationship, and the relationship between skin homogeneity and face attractiveness increase is a logarithmic function (Experiment 2A). These findings imply that people’s judgments of changes in faces and changes in attractiveness of faces involve in two different psychological processes, even though both of their changes result from manipulation of the homogeneity of the face’s skin.

The decision “different” requires a judgment accessible to consciousness while the decision “attractive” does not. People are extremely sensitive to facial attractiveness because of its importance in evolution and social signals. Facial attractiveness is evaluated in a rapid and automatic manner (Nakamura et al., 2017). Previous studies have shown that facial attractiveness can be processed even in the absence of conscious awareness (Hung et al., 2016; Nakamura and Kawabata, 2018). The attractiveness judgments activate a widely distributed neural network involving perception, decision, and reward circuits (Chatterjee et al., 2009). In particular, the orbitofrontal cortex (OFC) is one of the most prominent regions that have been proven to involve attractiveness judgments (Wang et al., 2015; Luo et al., 2019; Yarosh, 2019). It is a brain region that plays a role in reward value. Ishizu and Zeki (2013) investigated differences in the brain systems involved in aesthetic judgments and perceptual judgments (brightness judgments). They found that aesthetic judgments mobilized cortical and subcortical pathways that were not engaged in perceptual judgment. And OFC was more active in aesthetic judgment than perceptual judgments. OFC was also proved to be crucial to intuitive judgments without a conscious awareness (Volz et al., 2008; Horr et al., 2014). These pieces of evidence show that people can make “more attractive” judgments unconsciously.

Facial skin homogeneity had a significant effect on face attractiveness judgments. As the skin became more homogenous, the rate of participants choosing the beautified face to be more attractive than the original face increased. And there was a logarithmic relationship between the two. Even at the low level of beautification (30% beautification), the rates of the beautified face being more beautiful than the original face was higher than the baseline 50%. Thus, just a small increase in skin homogeneity could improve attractiveness. And, it can be seen in the logarithmic trend that as the level of skin beautification increases, the increase in the “more attractive” rates is more and more flat. When the level of skin beautification increased from 45 to 100%, perceived face attractiveness did not improve much. As the skin became more homogenous, its positive effect on attractiveness gradually diminished.

Our results showed that participants can perceive the weakly beautified face more attractive than the original version, although they tended to regard the two faces as physically same. Such sensitivity to facial attractiveness might be derived from the fact that facial skin homogeneity is an indicator of human youth, fertility, and health (Yarosh, 2019). In our facial skin and our perceptual system’s evolution, perceiving attractiveness may have become a strong physiological cue to help us make good judgment. Besides, facial skin homogeneity-based facial attractiveness perception can also be valid to of a person’s positive trait impressions, such as trustworthiness and competence (Jones et al., 2012; Tsankova and Kappas, 2016), although facial skin homogeneity looks merely like simple physical discrimination.

The “different” rate under the low beautification level (i.e., 30%) was significantly higher than that under the 0% beautification level, which indicated that the beautification operation was effective, and the participants were more able to find the difference between the two faces. However, this rate was still below 50%, which showed that the participants still tended to think that the two faces were the same. Under the 45% beautification level, the “different” rate was close to 50%, a tipping point at which participants’ judgments were ambiguous, with no obvious inclination. When the beautification level reached 100%, the participants could clearly realize that the original face and the beautified face were different.

We manipulated face skin homogeneity in this study. If the preference for homogeneous facial skin resulted from a sensory bias, a detectable difference in homogeneity between two otherwise-identical faces should change their perceived attractiveness. We found that a small increase in the homogeneity of facial skin resulted in a significant increase in the perceived attractiveness of the face. Similar results were also found in previous studies. Samson et al. (2010b) performed texture removal operations on female faces aged 45–65 in increments of 20% to form faces with varying degrees of homogeneous texture. They performed a forced choice task and found that a 20% change in facial skin had a significant positive effect on attractiveness. There was a similar effect on the adjustment of skin color homogeneity, with studies showing that faces with 25% smoother skin color appeared younger and healthier (Samson et al., 2011). Changes in the facial skin in our study were more subtle than in theirs, because the face stimulus used in this study was all young faces with fewer wrinkles and spots on the face. In such cases, minimal beautification can significantly improve perceived attractiveness (even in Experiment 2C using the least sensitive measurement technique). This suggests that when there are small changes in the skin condition of the face, people’s attractiveness perception for it is more sensitive than previously thought.

While a small increase in skin homogeneity can have a positive effect on attractiveness, there are limits to this effect. Samson et al. (2011) performed more than 50% skin homogenization on the faces without causing more variability in their health and age estimates. Similar results were found in this study. Our results showed a logarithmic trend in attractiveness changes. We found that with the increase of skin homogeneity, the attractiveness first increased greatly, and then the improvement of attractiveness became more and more gradual. Skin beautification to 100% is not the limit of skin homogeneity, it is just a percentage set for a specific beautification operation. We speculate that as skin homogeneity continues to improve, the increase in attractiveness will become smaller and may not even increase.

Our findings are consistent with a preference for homogeneous skin, as well as in previous studies, which reflect an innate or acquired preference for reliable skin cues for health and mate choice. Facial skin condition is a rapidly changing health signal that provides useful information on an individual’s current health and physiological state (Stephen et al., 2011). Some researchers believe that when the immune defense system is compromised, people are more vulnerable to microbes and parasites, which may be reflected in the surface texture of the facial skin (Fink et al., 2001). Skin texture is also suggestive of fertility (Fink et al., 2001), and dermatological studies have found that skin disorders are associated with elevated levels of sex hormones. Specifically, women with elevated androgen levels have more severe skin problems (Lucky, 1995; Karrer-Voegeli et al., 2009). In addition, with age, due to the progressive damage of the skin tissue, the skin gradually loses its elasticity, the pigmentation increases, and the skin homogeneity decreases (Robert et al., 2012). In conclusion, homogeneity of facial skin color and texture indicates that the individual is young, free of skin diseases, physically healthy, and has healthy levels of reproductive hormones.

For the tasks in this study, working memory of facial information was necessary. Participants were instructed to contrast the currently displayed face with the recently presented face that they had previously remembered. Visual perception and visual working memory work in tandem, and both share a common basis for face representation (Lee et al., 2012; Serences, 2016; Chang et al., 2017). Chang et al. (2017) also found that face representations in working memory contain sufficient image detail. This is the perceptual basis on which participants in this study can detect physical differences and attractiveness differences between two faces. Compared to the direct visual perception of presented faces, face representations in memory lose some information. For example, facial identity information decreases rapidly during the working memory period (Bae, 2020). Therefore, participants might ignore subtle changes in facial texture and regard the low-level beautified face identical to the original face.

It might be still unclear that which factors, discriminative ability or decision biases, are responsible for variations in object attribute perception. The skin homogeneity changes in this current study were all on young faces aged 17–18. Our participants were all around 20 years old. Their judgments of these faces’ attractiveness are not necessarily generalizable. It may vary by the age of the observer and the observed (He et al., 2021). Participants viewed an image instead of a person. Also, our study used face stimuli in a single race, which is Asian. The scope of interpretation of the findings needs to be extended. Finally, changes detection in the attractiveness of faces in our experiments does not completely rule out the effects on judgments of physical changes. Subsequent research needs to exclude the influence of different experimental paradigms, separate people’s judgments of physical changes of faces from those of face attractiveness to reduce confusion.

In summary, this study showed that along with the change of face skin homogeneity, individuals showed different sensitivities to the physical changes and perceived attractiveness changes from faces. It implies that people can magnify their attractiveness perception with the improvement of the face skin state even that they do not feel the physical changes of the faces. Our findings can contribute as a guide to the enhanced attractiveness with very slight skin improvements, thereby leading to proper grooming for skin care and base makeup.

## Data availability statement

The raw data supporting the conclusions of this article will be made available by the authors, without undue reservation.

## Ethics statement

The studies involving human participants were reviewed and approved by Institutional Review Board at Zhejiang Sci-Tech University. The patients/participants provided their written informed consent to participate in this study.

## Author contributions

All authors participated in designing the study. XZ and ZW programmed the data. XZ collected the data. Y-HS, XZ, and NL performed the statistics. XZ, NL, and Y-HS wrote the manuscript.
